# The lognormal handwriter: learning, performing, and declining

**DOI:** 10.3389/fpsyg.2013.00945

**Published:** 2013-12-19

**Authors:** Réjean Plamondon, Christian O'Reilly, Céline Rémi, Thérésa Duval

**Affiliations:** ^1^Laboratoire Scribens, Département de Génie Électrique, École Polytechnique de MontréalMontréal, QC, Canada; ^2^Département de psychiatrie, Université de MontréalMontréal, QC, Canada; ^3^Département de Mathématiques et Informatique, LAMIA, Université des Antilles et de la Guyanne, Campus de FouillolePointe-à-Pitre, Guadeloupe, France

**Keywords:** kinematic theory, lognormal models, handwriting analysis and generation, neuromuscular systems, learning, aging, lognormality

## Abstract

The generation of handwriting is a complex neuromotor skill requiring the interaction of many cognitive processes. It aims at producing a message to be imprinted as an ink trace left on a writing medium. The generated trajectory of the pen tip is made up of strokes superimposed over time. The Kinematic Theory of rapid human movements and its family of lognormal models provide analytical representations of these strokes, often considered as the basic unit of handwriting. This paradigm has not only been experimentally confirmed in numerous predictive and physiologically significant tests but it has also been shown to be the ideal mathematical description for the impulse response of a neuromuscular system. This latter demonstration suggests that the lognormality of the velocity patterns can be interpreted as reflecting the behavior of subjects who are in perfect control of their movements. To illustrate this interpretation, we present a short overview of the main concepts behind the Kinematic Theory and briefly describe how its models can be exploited, using various software tools, to investigate these ideal lognormal behaviors. We emphasize that the parameters extracted during various tasks can be used to analyze some underlying processes associated with their realization. To investigate the operational convergence hypothesis, we report on two original studies. First, we focus on the early steps of the motor learning process as seen as a converging behavior toward the production of more precise lognormal patterns as young children practicing handwriting start to become more fluent writers. Second, we illustrate how aging affects handwriting by pointing out the increasing departure from the ideal lognormal behavior as the control of the fine motricity begins to decline. Overall, the paper highlights this developmental process of merging toward a lognormal behavior with learning, mastering this behavior to succeed in performing a given task, and then gradually deviating from it with aging.

## Introduction

The generation of handwriting is a very complex neuromotor skill requiring the interaction of many cognitive processes. It aims at imprinting on a writing medium a message encoded in the trajectory of a pen tip, a trajectory ultimately made up of strokes superimposed over time (Thomassen et al., 1983; Kao et al., 1986; Plamondon et al., 1989; Van Galen, 1991). Taking a simplified sequential process approach (Plamondon et al., 1999), the production of a message can be seen as requiring the realization of numerous and diverse cognitive tasks. Starting from communication intents where the content of a message is elaborated, some semantic or cognitive networks must define and delimit the context of this message. Then various syntactic processes must activate the proper set of linguistic rules and interact with the specific lexicon of the selected language. The handwritten versions of the words in these lexicons are considered as being made up of allographs or character models. These models can be seen as ideal action plans that have been learned over the years. They can be instantiated to activate specific neuromuscular networks, producing a series of basic strokes superimposed over time– the fundamental units of handwriting movements—making up the intended pen tip trajectory.

The challenging and interesting element of handwriting studies is that they can be tracked from a broad spectrum of perspectives, from investigating high-level neurocognitive capabilities associated with language production down to the purely mechanical understanding of motor control gestures (Kandel et al., 2006; Bosga-Stork et al., 2011). Numerous researchers from various disciplines^1^ have been involved in studying some of these processes: neuroscience, experimental psychology, computer science and engineering, physics, education and developmental sciences, robotics, forensic science, paleontology and more. These studies were conducted using bottom-up or top-down approaches and exploit more or less sophisticated technologies, from complex fMRI investigations (Katanoda et al., 2001; Seitz, 2009; Richards et al., 2011; Shah et al., 2013) to simple non-invasive digitizer or instrumented pen signal analysis (Maarse, 1987; Baron and Plamondon, 1989; Teulings, 1996). Several studies deal with the fundamental understanding of the underlying neurocognitive and neuromotor processes (Thomassen and Teulings, 1983; Van Galen and Teulings, 1983; Van Galen, 1991) while others are primarily concerned with practical applications like on-line or off-line handwriting recognition (Nouboud and Plamondon, 1990; Plamondon et al., 1999; Plamondon and Srihari, 2000; Koerich et al., 2003; Lorigo and Govindaraju, 2006; Srihari et al., 2007; Tagougui et al., 2011), signature verification (Plamondon and Lorette, 1989; Leclerc and Plamondon, 1994; Pirlo and Impedovo, 2008; Impedovo et al., 2012; Plamondon et al., 2013a, 2014a), writer identification (Schomaker, 2007; Sreeraj and Idicula, 2011; Awaida and Mahmoud, 2012), and document analysis and processing (Doermann and Tombre, 2013). Many of these experiments rely in one way or another on a few basic models, and this is particularly true for investigations dealing with the motor control aspects of handwriting production. Indeed, many models have been proposed to study human movement control in general and handwriting in particular: models relying on neural networks (Bullock and Grossberg, 1988; Schomaker et al., 1989; Schomaker, 1991; Kalveram, 1998; Gangadhar et al., 2007), equilibrium point models (Feldman, 1966; Bizzi et al., 1978, 1992; Feldman and Latash, 2005), behavioral models (Thomassen et al., 1983; Van Galen and Teulings, 1983; Schmidt and Lee, 1999), coupled oscillator models (Hollerbach, 1981; Kelso, 1995; Zanone et al., 2005), emergent and convergent models (Plamondon, 1995a,b; Plamondon and Djioua, 2006), and models exploiting minimization principles (Wada and Kawato, 1995; Engelbrecht, 2001) including minimization of the acceleration (Neilson, 1993; Neilson and Neilson, 2005), of the energy (Nelson, 1983), of the time (Hermes and LaSalle, 1969; Enderle and Wolfe, 1987; Tanaka et al., 2006), of the jerk (Hogan, 1984; Flash and Hogan, 1985), of the snap (Edelman and Flash, 1987), of the torque changes (Uno et al., 1989) and of the sensory-motor noise (Harris and Wolpert, 1998). At the stroke level, many of these models exploit the properties of various analytic functions to reproduce and reconstruct human movements: exponentials (Plamondon and Lamarche, 1986), second order systems (Denier van der Gon and Thuring, 1965; Dooijes, 1983), Gaussians (Leclerc et al., 1992), beta functions (Alimi, 2003), splines (Morasso et al., 1983), and trigonometric functions (Hollerbach, 1981; Maarse, 1987).

Among the models providing analytical representations of the trajectories, the family of lognormal models predicted by the Kinematic Theory of rapid human movements (Plamondon, 1995a,b, 1998; Plamondon et al., 2003; Plamondon and Djioua, 2006) has been used to explain most of the basic phenomena reported in classic studies on human motor control (Plamondon and Alimi, 1997) and to study several factors involved in fine motricity (Djioua and Plamondon, 2009a; O'Reilly and Plamondon, 2011; Woch et al., 2011). Apart from these fundamental studies, these models have also been used, directly or indirectly, in many practical applications like the design of a signature verification system (Plamondon, 1994), the development of tools to help children learn handwriting (Carrières and Plamondon, 1994; Djeziri et al., 2002), the generation of synthetic signatures and gestures databases for algorithm testing or classifier learning (Almaksour et al., 2011; Galbally et al., 2012a,b) as well as the design of biomedical set-ups to detect fine motor control problems associated with brain stroke risk factors (O'Reilly and Plamondon, 2011, 2012a,b).

In this paper, we push one step further the lognormality concept to point out how it can be used to provide a global estimate of the performance of a handwriter. We begin by presenting a brief overview of the Kinematic Theory of rapid human movements to particularly illustrate how its family of lognormal models can be seen as describing human beings when they are in perfect control of their movements. We then give a list of software tools that we have developed over the years to automatically extract the model parameters from handwriting. Typical examples of original and reconstructed trajectory patterns are presented. References are also given to specific studies dealing with how the theory can be used to reveal the conditions for a lognormal handwriter to successfully execute a required trajectory. Continuing along this paradigm, we look at motor learning as a shift toward a lognormal behavior, a conduct that we then master and exploit for a large part of our life and that we slowly see decrease as we get older. To further investigate this interpretation of the lognormality, we look at some aspects of the move toward lognormality by analyzing the handwriting of young kindergarten children, 3 to 5 years old, to emphasize how they improve the control of their fine motricity as they perform typical learning lessons. Using a similar presentation scheme, we also study the move away from lognormality by analyzing aging effects on handwriting. Typical changes observed in the model-based descriptions are reported, illustrating the deviation from lognormal behavior. The paper concludes by briefly summarizing the results and exploring some possible extensions of this approach to study health problems (Parkinson disease, Alzheimer's disease, brain stroke) and rehabilitation therapy.

## Materials and methods

### Defining and taking advantage of lognormality

The Kinematic Theory describes a neuromuscular network involved in handwriting production as a linear system that controls the velocity of the pen tip. Assuming that such a system is made up of numerous coupled subsystems and that this coupling can be expressed with proportionality relationships between the cumulative time delays associated with the activation of these subsystems, the theory predicts, using the Central Limit Theorem, that the magnitude of the velocity profiles produced by the global system will tend toward a lognormal pattern (Plamondon, 1995a,b, 1998; Plamondon et al., 2003):

(1) $$\begin{array}{l} \left|{\vec{v}}_{i}(t;P_{i})\right|=D_{i}\Lambda_{i}(t_{0i};\mu_{i},\sigma_{i}^{2})\\ \;=\;\frac{D_{i}}{\sigma_{i}\sqrt{2\pi }(t-t_{0i})}\exp\text{​}\left[-\frac{{\left[\ln(t-t_{0i})-\mu_{i}\right]}^{2}}{2\sigma_{i}^{2}}\right] \end{array}$$

where the set of parameters *P_i_* = [*D_i_, t_0 i_*, μ*_i_*, σ*_i_*] describing a lognormal pulse refers to:

*D_i_*: the input command, which is the intended distance to be covered with the pulse;

*t_0 i_*: the time occurrence of that command, as instantiated in the central nervous system (CNS);

μ*_i_*: the log time delay (the time delay on a logarithmic time scale);

σ*_i_*: the log response time (the response time on a logarithmic time scale).

The production of a given stroke can thus, be seen as the process of recruiting a sufficient number of time-coupled neuromuscular units to produce the most perfect lognormal profile. These lognormal functions are the basic primitives, the elementary strokes that can be used to produce any complex pen tip trajectory. Under this paradigm, handwriting generation starts with the instantiation of an action plan made up of virtual targets linked together by circular arcs. This planning space, which can be modeled with a grid of leaky integrators simulating learning neurons (Plamondon and Privitera, 1995), acts as a command generator, each command producing a lognormal stroke. A sequence of commands results in a vector summation process where the velocities of individual strokes are superimposed over time to produce a given pattern (a letter, a word, a signature, a gesture, and so forth) (Plamondon and Guerfali, 1998). The magnitude and direction of the velocity of a given trajectory are thus, described by a Sigma-Lognormal equation (Plamondon and Djioua, 2006):

(2) $$\begin{array}{l} \vec{v}(t)=\sum_{i=1}^{N}{\vec{v}}_{i}(t;t_{0i},\mu_{i},\sigma_{i}^{2})\\ =\sum_{i=1}^{N}D_{i}\left[\begin{array}{l} \cos(\theta_{i}(t))\\ \sin(\theta_{i}(t)) \end{array}\right]\Lambda_{i}((t;t_{0i},\mu_{i},\sigma_{i}^{2});N\geq 2 \end{array}$$

(3) $$\theta_{i}(t)=\theta_{si}+\frac{\left(\theta_{ei}-\theta_{si}\right)}{2}\left[1+erf\left(\frac{\ln(t-t_{0i})-\mu_{i}}{\sigma_{i}\sqrt{2}}\right)\right]$$

where θ*_si_* and θ*_ei_* stand, respectively, for the starting and ending angular direction of each discontinuous stroke, as ideally represented in the action plan. When only straight line movements are produced, Equation 2 reduces to what is called a Delta-Lognormal equation:

(4) $$v\left(t\right)=D_{1}\Lambda \left(t;t_{0},\mu_{1},\sigma_{1}^{2}\right)-D_{2}\Lambda \left(t;t_{0},\mu_{2},\sigma_{2}^{2}\right)$$

where the subscripts 1 and 2 refer, respectively, to the agonist and antagonist neuromuscular networks.

From a validation point of view, apart from its outstanding performances in reproducing handwriting under various conditions (Plamondon et al., 1993, 2014a; Alimi and Plamondon, 1994; Plamondon and Alimi, 1997; Feng et al., 2002), as well as making clear predictions regarding the conditions under which the various speed-accuracy trade-offs emerge (Plamondon and Alimi, 1997)^2^, two decisive experiments have provided functional and biomedical supports to the theory and confirmed its underlying hypotheses and its physiological significance. On the one hand, to get a lognormal convergence, the subsystems constituting a neuromuscular network must be synchronized in such a way that the cumulative time delays of the command propagating along a given network must obey proportionality relationships. These proportional effects have been clearly observed between various pairs of upper arm muscles involved in the production of rapid movements using electromyography (EMG) (Plamondon et al., 2013b). On the other hand, when a lognormal is observed, the theory presumes that it is the result of a command that has been activated in the CNS at a given time *t*_0_ (see Equation 1). This prediction has also been confirmed using electroencephalography (EEG), where it has been shown that a specific evoked response potential (ERP) was produced at *t*_0_ (O'Reilly et al., 2013).

**Figure 1:**
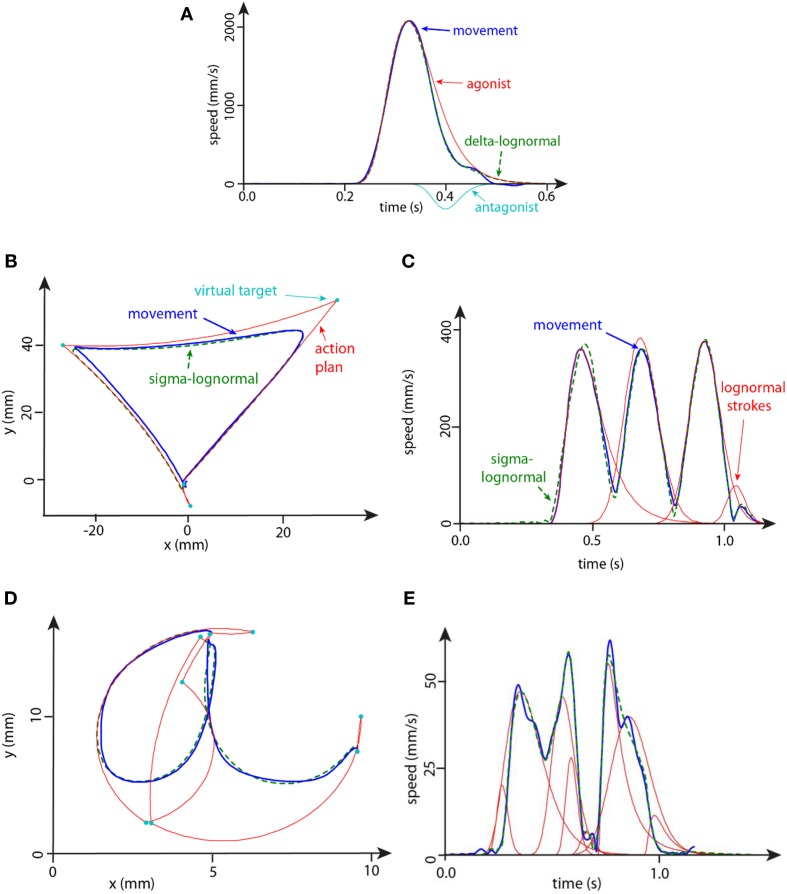
**(A)** Example of the speed profile of a delta-lognormal modeling for a typical fast reaching movement. **(B,C)** Example of the trace **(B)** and the speed profile **(C)** of the sigma-lognormal modeling of a typical triangular movement. **(D,E)** Example of the trace **(D)** and the speed profile **(E)** of the sigma-lognormal modeling of a typical handwritten letter “a.”

Several software packages have been developed over the years to extract the lognormal parameters from various velocity curves under different experimental data acquisition conditions and set-ups (Guerfali and Plamondon, 1998; Djioua et al., 2007; Plamondon et al., 2007; Djioua and Plamondon, 2009b; O'Reilly and Plamondon, 2009a, 2010, 2012c), and most up-to-date algorithms have been implemented in ScriptStudio research software. These different tools allow a researcher to study handwriting through the parametric lognormal description of the neuromuscular networks involved in a given task (Plamondon et al., 2009; Plamondon, 2013). Starting with the pen tip position [x(*t*), y(*t*)] as sampled by a digitizer, these software tools compute the velocity vector and, using different optimization algorithms, extract the lognormal parameters of the strokes that best describe the sampled trajectory. Figure 1 presents three typical examples of velocity reconstruction. In 1A, a single stroke is reconstructed using a Delta-Lognormal equation (Equation 4), while in 1B a complex graphic trace has been reconstructed using a Sigma-Lognormal equation (Equations 2 and 3) resulting in the speed profile shown in 1C. In 1D and 1E, similar patterns are shown for the handwritten character “a.” In each case, the parameter extraction algorithm provides the values of the lognormal parameters that have been found to automatically reconstruct a profile, the number of lognormals (nbLog) required to reconstruct the signal as well as a measure of the quality of the reconstruction as evaluated by computing the signal-to-noise ratio (SNR) between the original ${\vec{v}}_{o}(t)$ and the reconstructed ${\vec{v}}_{r}(t)$ profiles:

(5) $$\text{SNR}=10\log\text{​}\left(\frac{\int_{t_{s}}^{t_{e}}\left[v_{ox}^{2}(t)+v_{oy}^{2}(t)\right]dt}{\int_{t_{s}}^{t_{e}}\left[{\left(v_{ox}(t)-v_{rx}(t)\right)}^{2}+{\left(v_{oy}(t)-v_{ry}(t)\right)}^{2}\right]dt}\right)$$

where the global effect of the distortions is computed using the velocity components *v_x_* (*t*) and *v_y_* (*t*) from the beginning *t_s_* to the end *t_e_* of these Cartesian signals.

In other words, with these optimization tools, for each handwriting trace produced by a subject, the Kinematic Theory provides a new parametric representation space to study its motor control behavior. This offers a new window into studying human movements, where some specific strategies for succeeding at a given task can be pointed out, these strategies relying on how the lognormality is mastered and exploited.

For example, it has been demonstrated that strong coupling of agonist and antagonist neuromuscular networks were necessary to produce a single fast stroke with a direction reversal without a pause at the breaking point (Woch and Plamondon, 2010). This resulted in very high correlations between the agonist and antagonist lognormal parameters extracted from each individual trajectory (Woch et al., 2011). Similarly, analyzing a Fitts' task under the paradigm of the Kinematic Theory, it has been shown that the subjects had to correlate more tightly the impulse commands sent to the agonist and antagonist neuromuscular systems in order to achieve good performances as the difficulty of the task increases whereas the correlation in the timing of the neuromuscular action co-varied with the size of the trajectory's geometrical properties (O'Reilly and Plamondon, 2013).

Overall, the theory has not only been experimentally supported in numerous predictive and physiologically significant tests but it has also been shown to be the ideal mathematical model to describe the impulse response of a neuromuscular system (Djioua and Plamondon, 2010), which results in what is known as “asymmetric bell-shaped velocity profiles.” This mathematical demonstration suggests that the asymptotic convergence toward lognormal impulse responses and velocity patterns can be interpreted as reflecting the behavior of subjects who are in total control of their movements. In this context, if we specifically focus on the basic mathematical convergence toward lognormality, handwriting learning, on the one hand, can be interpreted as a migration toward an ideal control of perfectly mastered movements. In other words, we can account for the lognormality of the underlying neuromotor processes as a convergence toward the complete mastering of a given task. On the other hand, aging should reveal a progressive decrease of the fine motor control, as reflected by a departure from lognormality.

Additionally, from a mathematical point of view, the Kinematic Theory is a theory of convergence toward smoothness. The lognormal function is an optimal descriptor of the velocity profiles: the smoothest velocity being reached when the energy associated with the convergence error toward lognormality is minimized (Djioua and Plamondon, 2010). As such, the Kinematic Theory can be considered as an ultimate minimization theory. We strengthen this statement in the following sections by investigating two phases of this process: characterizing handwriting learning as a move toward lognormality and characterizing aging as a move away from lognormality. To do so, we utilize the Kinematic Theory to reconstruct various handwriting samples and analyze them using three performance criteria: the SNR of the reconstructed pattern as defined in Equation 5, the nbLog used to make that reconstruction, and the SNR/nbLog ratio which can be seen as a global indicator of a given writer's motor control skills.

### Experiment 1: learning

#### Moving toward lognormality

The mastering of fast writing and drawing movements is the norm in healthy adults with full control of their neuromuscular system (Zesiger, 1995; Karldottir, 1996; Senatore and Marcelli, 2012), but this is not the case for young children. According to many studies, the automation of handwriting skill by children is the usual outcome of a non-monotonous learning phase which takes around 10 years (Meulenbroek and Van Galen, 1988; Vinter and Mounoud, 1991; Albaret and Santamaria, 1996; Zesiger et al., 2000; Frélicot et al., 2002; Chartrel and Vinter, 2004). In the context of the Kinematic Theory, it is after this period that lognormality should be well established and should begin to be fully exploited. Indeed, it has been shown that rapid movements produced by young adults can be almost perfectly modeled as sums of lognormal vectors (Guerfali and Plamondon, 1998; O'Reilly and Plamondon, 2009a,b). When children are still in the early steps of their learning process, is it expected that a lognormal behavior should be already discernible.

The goal of the present study was to determine if the SNR of the reconstructed pattern, the nbLog used to make a reconstruction and the SNR/nbLog ratio could be good indicators of the progress of children's performances in early phases of scolarship (PS, MS, GS).

#### Participants

The subjects that have been chosen for this preliminary study had reached the best scriptural behavior according to three early grade criteria of kindergarten, and as such were considered as good writers according to their grade. Such choice is necessary to try to limit *a priori* the effects of some factors that can influence a child's handwriting skills like handwriting learning difficulties. To select such samples of children for each school level we proceeded as follows: during the 2011–2012 school year, a preliminary group of 66 pupils was randomly selected within the population of a Guadeloupe kindergarten to take part in preliminary data acquisition. These children were from three school grades: the PS level was grouping 3- and 4-year-old participants; the MS level, the 4- and 5-year-olds and the GS level, the 5- and 6-year-olds. The members of the PS, MS, and GS grades had 6, 18, and 30 months of handwriting lessons, respectively. This preliminary study was necessary for screening, from a large pool of possible tasks, those that had the higher discrimination potential. However, data collected at this stage were not adequate for statistical analysis since only a few samples, generally three to five, could be collected for each kind of movement, to avoid exposing young children to large number of trials. Given the high variability of human motor productions, such a small number of repetitions per children and per movement types was not sufficient to provide stable averages of children neuromotor characteristics.

Thereafter, for each of the three grades, five children were selected from this first group as participants in the present study. This selection was performed according to three inclusion criteria. First, the child's teacher had pointed out that in normal lessons the child was interested in handwriting and drawing, and had demonstrated satisfying performances in these activities. Second, the teacher had certified that the child had completed the two drawing and the two writing activities proposed to all the children in the group during normal classroom hours. These first two criteria confirmed that each child's performance was in compliance with the French kindergarten program expectations for their specific group level. The third criterion dealt with the results of the analysis of the child's performance at the first data acquisition phase. The teacher and the experimenter had found that the child was motivated to take part in the experiment and that the child had produced all the required trajectories (about 40). The different trajectories that the child had produced for the first data acquisition had been reconstructed on average with a SNR >25 dB and with an optimal nbLog for a given pattern consistent with the ideal sigma-lognormal model. This second experimental stage was necessary for producing data that are adequate for statistical modeling (i.e., a large number of repetitions per children for a small subset of highly discriminative tasks). For ethical reasons and given the repetitive (and possibly boring) nature of this experiment performed by very young children, only the most interested children had to be selected, resulting in a reduction from the initial pool of 66 children to a subset of 15 highly interested children for the second stage.

For each of these three groups (GS, MS, and PS), the five selected participants were thus, *a priori* considered as being able to successfully execute the required tasks and as having the most stable level of performance with respect to their own level of learning and expertise.

#### Procedure and apparatus

During the first acquisition phase, selected children were asked to produce their movements on a Wacom Intuos3 tablet and the kinematic of the motion was digitized at 200 Hz. Eleven patterns (Duval, 2012) had to be performed: an oblique trace, five pseudo-letters, and three cursive letters (l, p, and r). It must be noted that each child prior to participating to the study had been given the chance to familiarize himself or herself with the equipment, practicing the required patterns under the supervision of a teacher.

A conclusion of the first acquisition phase was that among the 11 tested patterns, the most successful ones were the oblique traces (/) and the bridge movements (∩) (Duval et al., 2013). Thus, these two tasks were selected for the next stage that aimed to investigate a potential migration toward lognormality. For this second acquisition phase, the requirement was to rapidly produce an oblique trajectory, starting from a common origin and reaching a given fixed target, and a bridge trace, starting from a common origin and reaching a given fixed target after passing over an intermediate target. Each pair of trajectories was repeated 30 times by every participant, except for a few very young PS participants who had difficulty staying concentrated on the task. Due to these difficulties among the youngest children, 25 movements were missing. A few movements (8) were also lost, having been inadvertently destroyed by the experimenter during the acquisition.

#### Signal processing and statistical analyses

Each x(*t*) and y(*t*) trajectory produced by a participant was input into the ScriptStudio software package for automatic lognormal segmentation and optimal parameter extraction. These parameters were then used to reconstruct the velocity profile of the original trajectory. The three performance criteria (SNR, nbLog, and SNR/nbLog) were computed from this reconstruction process. To observe if some of the performance criteria could show differences between young writers of GS, MS, and PS, we tested whether our three criteria were uniformly distributed in the different classes of writers (GS, MS, and PS), answering each of the following questions:

(Q1) Is the quality of reconstruction (SNR) evenly distributed among all classes of writers?
(Q2) Is average of the nbLog the same for all classes of writers?
(Q3) Is average of the ratio SNR/nbLog the same for all classes of writers?
(Q4) Is average of the SNR the same for all classes of writers?

We have analyzed the distribution of SNR in a contingency table and the averages of nbLog, of the ratio SNR/nbLog and SNR on our raw data.

### Experiment 2: aging

#### Moving away from lognormality

Numerous experiments have shown that movements become slower and less coordinated when people get older (Contreras-Vidal et al., 1998; Ketcham and Stelmach, 2004; Barry et al., 2005; Robinovitch et al., 2005) but it is still unclear if this is due to a motor system deterioration or if it is the result of compensatory strategies (Latash and Anson, 1996; Heuninckx et al., 2008). In a previous study (Woch et al., 2011), we asked seven subjects aged 63 to 70 and seven subjects aged 26 to 29 to produce handwriting strokes on a digitizer, in response to an audio stimulus. The subjects were instructed to make bidirectional strokes [i.e., delta-lognormal strokes that exhibit a significant return in their trajectory (Woch and Plamondon, 2010)] as fast as possible and with their dominant hand. Three of the older subjects did not produce enough bidirectional primitives reconstructed with a SNR >15 dB^3^ and were excluded from the subsequent data analysis. This investigation exposed a substantial increase in neuromuscular response delays and a decrease in command amplitudes with age. Both the agonist and antagonist systems were similarly affected. Furthermore, it was observed that age had a proportional effect on the various time characteristics of the movements. Among other things, this experiment pointed out that, even in the case of a significant slowing down of the neuromuscular systems, the elderly could still achieve optimal movement responses, characterized by the reconstruction of their gestures with a single delta-lognormal primitive, similar to those produced by young healthy subjects. The number of successful attempts was smaller in the older group. These preliminary results indicated a mathematical depiction of age-related movement alterations.

In the present study, we used reaching movements and triangular drawings to illustrate how aging phenomena affect handwriting, pointing out the increasing departure from the ideal lognormal behavior as the control of fine motricity begins to decline. In this experiment, reaching movements are investigated because they are one of the most elementary types of movement normally involving a single stroke. They can be modeled using a delta-lognormal function. These delta-lognormal movements are considered as a fundamental primitive used in synergies to compose more complex patterns such as those used in handwriting and in drawing. As for the triangular movements, they are investigated because they constitute a relatively simple task requiring the coordination of at least four stroke primitives, each one described by a single lognormal (see Figure 1C).

#### Participants

**Figure 2:**
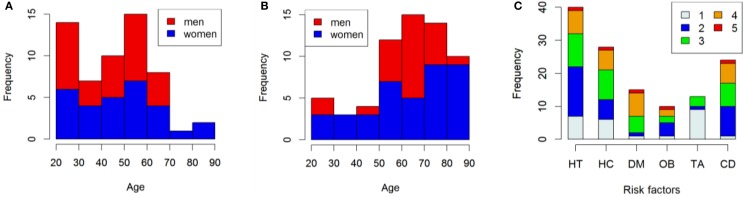
**(A)** Distribution of age and gender for the NRF subsample. **(B)** Idem, but for the WRF subsample. **(C)** Distribution of the risk factors in the WRF subsample. The height of the bars shows the overall number of subjects with each risk factor. As subjects may have more than one risk factor simultaneously, the bars are separated into colored sections indicating how many risk factors the subjects have. For example, 40 subjects have HT (height of the HT bar); among these, about 8 subjects have only the HT (the light gray portion of the HT bar) whereas one subject who has HT also has 4 other risk factors, for a total of 5 (the red portion of the HT bar).

Two participant samples were studied to analyze the effect of age in healthy and less healthy populations. The first sample (hereafter labeled NRF for “no risk factor”) contained 29 women and 28 men, with age varying between 25 and 87 years old. It corresponds to the control subset of a sample of 120 subjects which participated in a study on the impact of brain stroke risk factors on movement kinematics (O'Reilly and Plamondon, 2010, 2011, 2013). The age distribution of the considered subsamples can be seen in Figure 2A. Participants were considered healthy, had no brain stroke history, and had none of the following brain stroke risk factors: alcoholism, cigarette smoking (CS), obesity (OB), hypertension (HT), hypercholesterolemia (HC), cardiac disease (CD), and diabetes (DM). The second sample (hereafter labeled WRF for “with risk factor”) was constituted of 39 women and 24 men, with age also varying between 25 and 87 years old. Each of these participants had at least one of the previously listed risk factors, except for alcoholism which was reported by none of the subjects. The age and gender distribution as well as the risk factor distribution within this sample can be seen, respectively, in Figures 2B,C.

As can be appreciated in Figures 2A,B, the distribution of gender and age appears to be distributed independently. This is confirmed by Kruskal–Wallis tests showing that the average age of men and women is not statistically different in both samples (NRF: *W* = 344, *p* = 0.3258; WRF: *W* = 412, *p* = 0.4319). Thus, gender is omitted in the following analyses since the effect of age and gender cannot be confounded, the gender being reasonably balanced with respect to age.

Participants in both samples were volunteers from the École Polytechnique community or patients from Hôpital De Réadaptation Villa Medica. They all gave informed written consent. The experimental protocol was approved by the Ethics Boards of École Polytechnique and Hôpital de Réadaptation Villa Medica.

#### Procedure and apparatus

**Figure 3:**
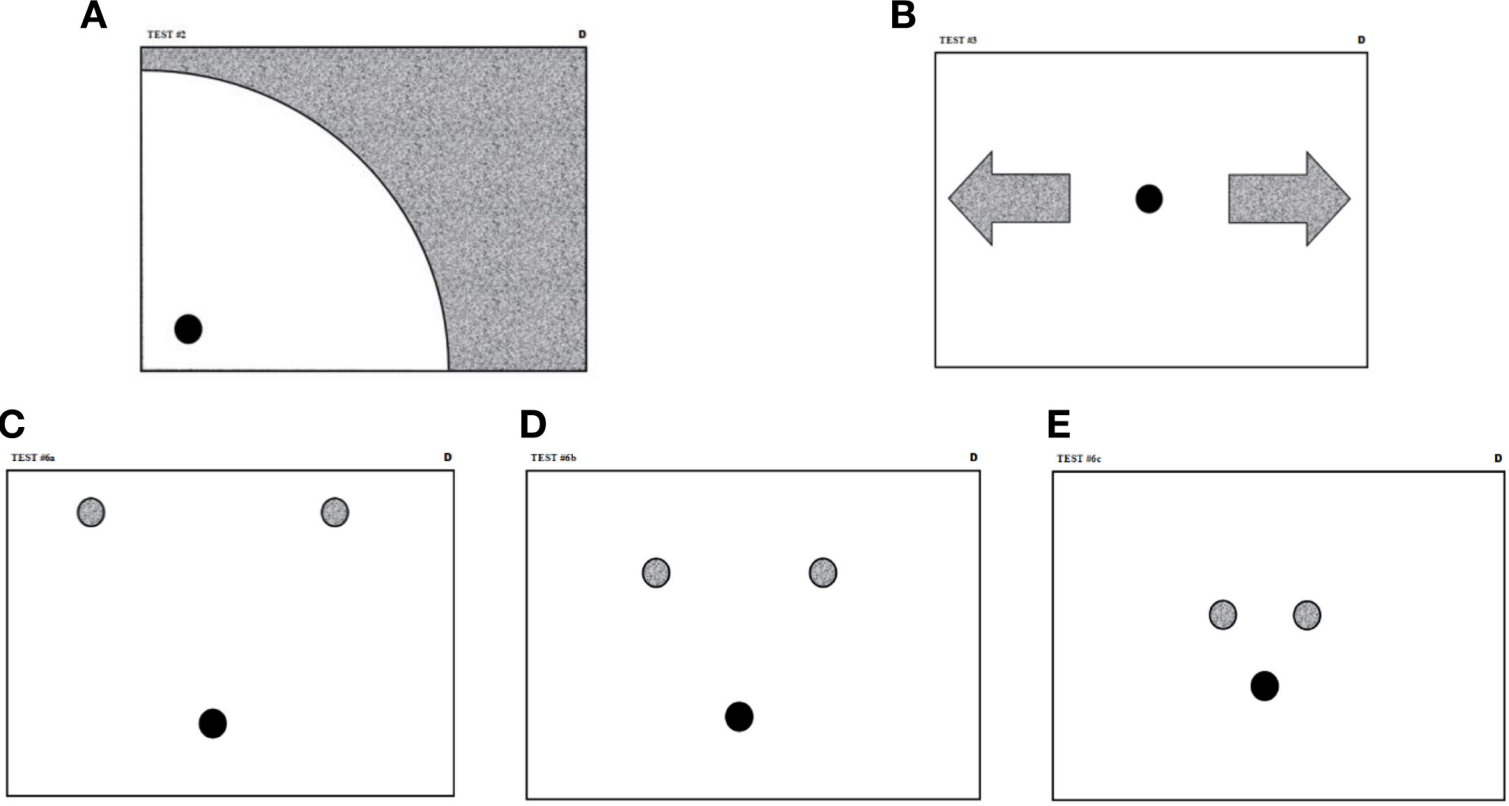
**Guiding sheets used for the simple reaction time experiments (A), the choice reaction time protocol **(B)**, and the triangular drawings **(C–E)**.** The starting position is shown as a dark circle and the target zones as gray areas. For triangular drawing sheets, the targets are 15 mm in diameter and are positioned at the apexes of equilateral triangles with vertexes of 135 mm **(C)**, 90 mm **(D)**, and 45 mm **(E)** long.

Participants were submitted to a test battery of nine experiments. The full experimental protocol can be seen in (O'Reilly, 2012; O'Reilly and Plamondon, 2012b; Plamondon et al., 2014b). For the present analysis, we considered two sets of acquisitions. The first one contains the data of three reaction time experiments, two on simple stimulus (auditory and visual) and one on choice stimulus (visual). A targeted number of 15 valid samples were collected for the tasks using simple stimulus and 30 for the choice stimulus. The choice stimuli were leftward and rightward arrows—chosen at random—indicating the requested direction for the reaching movement. The targeted zones were very large such that no precision was required. The movement had to be performed on a Wacom Intuos2 tablet and the kinematic of the motion was digitized at 200 Hz. Figures 3A,B show the sheets that were placed under the transparent folding of the tablet to guide the subjects. A movement amplitude of at least 130 mm was asked for in reaction to simple stimuli, 38 mm for choice stimuli. The laps of time between the instant the subject took place at the starting position and the emission of the stimulus was randomly distributed following a flat hazard function (i.e., the exponential distribution), with delays varying between 0 and 10 s.

The second set of movements consisted of triangular drawings arranged in a factorial design, with three triangle sizes, two drawing orientations, and two repetitions. Triangles were constituted of a series of three targets to be hit sequentially and in a continuous trajectory with the movement coming to a full stop within the last target. Figures 3C–E shows the guiding sheets that were used for this task.

#### Signal processing and statistical analyses

For fast movements performed in response to stimuli, such as in reaction time tests, the delta-lognormal (Equation 4) is the most appropriate type of modeling and has thus, been adopted for this investigation. The system proposed by (Djioua and Plamondon, 2009b) cascaded with a supplementary non-linear optimization step was used to obtain the delta-lognormal parameters from the digitized movements. To test the hypothesis that, with normal aging, motor performances migrate away from a lognormal behavior, we studied the relationship between the age and the SNR of the delta-lognormal reconstruction. Since the nbLog is fixed *de facto* to two components in a delta-lognormal model, the two other performance criteria (nbLog and SNR/nbLog) were not useful here.

For triangular movements, a sigma-lognormal modeling using the Robust X_0_ algorithm (O'Reilly and Plamondon, 2009a) was adopted. Our three response variables (SNR, nbLog, and SNR/nbLog) were studied in relation with the movement direction and size as well as with the subject age as descriptive variables. For statistical analysis, results of repetitions were averaged, giving six outcomes per subject following the factorial plan of three sizes by two orientations. All three response variables were modeled with linear regression using our three descriptive variables, as well as the interaction between the subjects' age and the interaction between the triangles' size and drawing orientation.

## Results

### Learning

#### On children's trend to migrate toward a lognormal behavior

**Figure 4:**
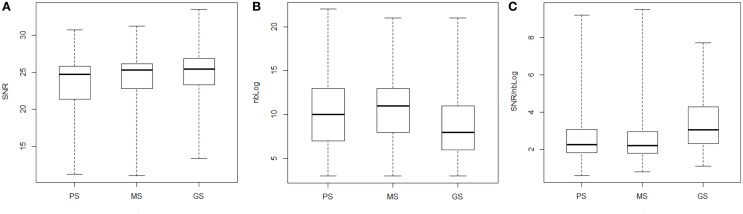
**SNR (A), nbLog (B), and SNR/nbLog ratio (C) as functions of the writer class for all movements**.

As seen in Figure 4A, for all oblique traces and bridges, the more advanced in the writing learning phase the writers are, the better is the reconstruction of their trajectories on average. To answer the question Q1 we performed the chi square test to check the hypothesis of independence between the SNR and the three different classes of writers χ^2^(2, *N* = 438) = 9.463. The result was significant (*p* = 0.009). So, we conclude that the quality of the reconstruction depends on the grade of writers A detailed comparison of the grade of writers per pairs showed, as reported in Table 1, that the quality of the reconstruction is dependent on the classes for the class of GS and PS χ^2^(1, *N* = 291) = 8.690, *p* = 0.003, respectively, MS and PS χ^2^(1, *N* = 288) = 4.919, *p* = 0.027 but it is independent of the grade for GS and MS χ^2^(1, *N* = 297) = 0.539, *p* = 0.463. Thus, the variable SNR can translate the migration to a lognormal behavior between writers groups PS and GS, and PS and MS.

**Table 1:** **Test on classes for the SNR for oblique traces**.

**SNR threshold 25 dB**	**GS-MS-PS**	**GS-MS**	**GS-PS**	**MS-PS**
Test statistic	9.463	0.539	8.690	4.919
Ddl	2	1	1	1
*p*-value	**0.009**	0.463	**0.003**	**0.027**
Critical value	5.991	3.841	3.841	3.841

Bold is used to indicate statistical significance (p-value <0.05). The chi square test was used to compare the distributions of the SNR.

To answer the question Q2 we performed the Kruskal–Wallis test to verify whether the average number of lognormals, nbLog, was the same in the three classes of writers. The results were significant as shown in Table 2 (*p*-value = 2.025e-21), indicating a difference in averages. To confirm these trends, we performed Mann–Witney tests to verify whether the nbLog was the same in the classes of writers taken in pairs (GS, MS), (GS, PS), and (MS, PS). As shown in the same Table 2, results indicate that averages were significantly different (all *p* < 0.01) when using an α = 0.05 corrected at 0.0083 for multiple comparisons, using the Bonferonni approach.

**Table 2:** **Test on classes nbLog, SNR/nbLog, and SNR for oblique traces**.

**Variable**	**nbLog**	**Kruskal–Wallis**	**SNR/nbLog**	**Kruskal–Wallis**	**SNR**	**Kruskal–Wallis**
	**(*p*-value)**	**chi-squared *W***	**(*p*-value)**	**chi-squared *W***	**(*p*-value)**	**chi-squared *W***
GS-MS-PS	**2.025e-21**	95.2973	**4.071e-11**	47.849	**8.6e-3**	14.116
GS-MS	**6.544e-10**	6471.5	3.809e-02	15659	0.1742	12031
GS-PS	**7.807e-21**	3285.5	**1.960e-08**	14604	**2.21e-4**	13225
MS-PS	**2.679e-05**	6538	**1.315e-02**	13532	**0.0165**	12058

Bold is used to indicate statistical significance (p-value <0.0166, corresponding to α = 0.05 corrected for multiple comparisons using Bonferonni). The Kruskal–Wallis test was used to compare several distributions, and the Mann Whitney test to compare two distributions.

To answer the question Q3 we performed the same tests to check whether the distribution of the SNR/nbLog ratio was similar in the three classes of writers on the one hand and in all the classes of writers compared by pair on the other hand. Results indicate that all distribution had significantly different averages (Table 2) for the three classes of writers considered together (*p*-values = 4.071e-11) as well as for each class taken by pair except for the GS-MS pair. Finally to answer the question Q4, we performed the same tests to check whether the distribution of the SNR was similar in the three classes of writers and in all the classes of writers compared by pair. Results indicate that the distributions had significantly different averages (Table 2) for the three classes of writers considered together (*p*-values = 8.60e-3) as well as for the pairs of classes GS-PS, MS-PS, (*p*-values = 2.21e-4/0.0165). Only the distribution of the SNR for GS and MS was similar (*p*-value = 0.1742), which suggests that after 1 year of handwriting learning in the PS group, the children have already made substantial progresses. When they reach the MS and the GS groups, the quality of the reconstruction of their trajectories become similar but still differ in term of the number of lognormal needed to reconstruct these. In short, the box plots of the Figures 4B,C show that for classes MS, GS, the more the writers advance in their writing learning phase, the more they tend control their lognormal behavior. That is, they use fewer lognormals to perform a trace and their ability to control their motor system, as assessed by the ratio SNR/nbLog, is improved.

### Aging

#### Reaching movements

**Figure 5:**
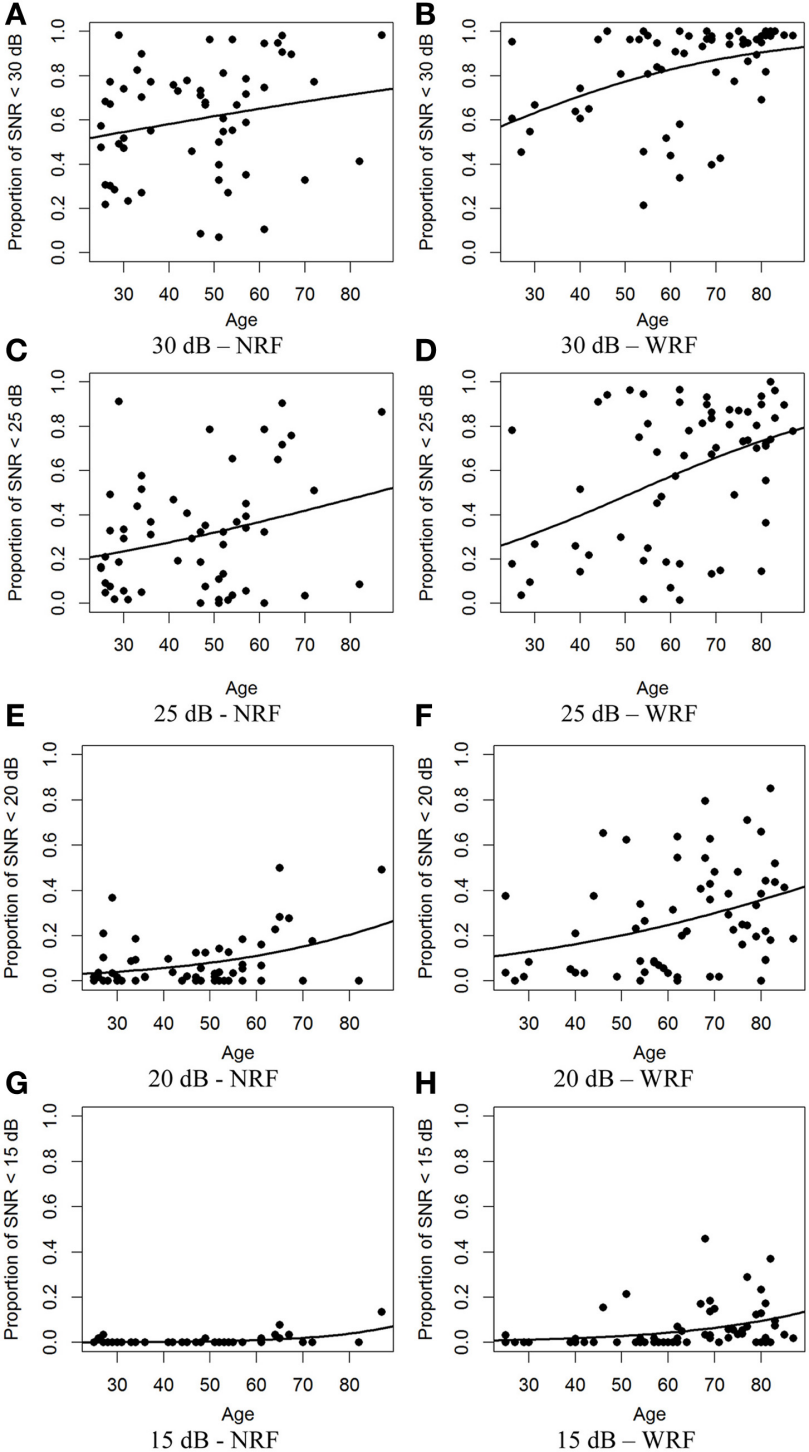
**Scatter plots of the proportion of rejected movements when considering the rejection criterion SNR < λ, for different values of λ (in dB).** A logistic regression curves has been added to the plots to show the average tendency. The plots (**A,C,E,G**) refer to the population with No Risk Factor (NRF) having a SNR < 30,25, 20 and 15 dB, respectively. The plots (**B,D,F,H**) refer to the population With Risk Factor (WRF) under the same SNR conditions.

Figure 5 shows, for both NRF and WRF samples, scatter plots displaying the relationship between the numbers of movements associated with a poorer delta-lognormal modeling according to the SNR < λ rule for the λ taken in decreasing order as 30, 25, 20, and 15 dB. In these graphs, a positive slope indicates that older participants have more difficulty producing neat delta-lognormal movements, suggesting poorer motor control.

A logistic regression was performed to model the variation of the proportion of low SNR movements as a function of the age. The best fitting curve was added on the plots of Figure 5. Modeling coefficients and associated *p*-values are reported in Table 3.

**Table 3:** **Modeling coefficients and associated *p*-values computed for logistic modeling of data shown in Figure 5**.

**SNR threshold**	**NRF**		**WRF**	
	**Intercept (*p*-value)**	**Age (*p*-value)**	**Intercept (*p*-value)**	**Age (*p*-value)**
30	**−0.2550 (2.59e-2)**	**0.01765 (9.00e-10)**	**−0.4873 (2.04e-3)**	**0.03440 (2.67e-38)**
25	**−1.8189 (2.38e-47)**	**0.02134 (1.30e-17)**	**−1.8462 (1.57e-37)**	**0.03578 (1.63e-56)**
20	**−4.2304 (4.12e-76)**	**0.03592 (2.52e-18)**	**−2.6934 (1.71e-52)**	**0.02640 (5.14e-24)**
15	**−8.5925 (1.02e-24)**	**0.06751 (1.42e-7)**	**−5.6344 (6.55e-41)**	**0.04233 (4.87e-13)**

Bold is used to indicate statistical significance (p-value <0.05).

As can be seen by the positive age coefficients in Table 3, regardless of the selected λ threshold, there is a significant overall increase of proportion of low-SNR movements with age. These data clearly support a positive relationship between age and the proportion of low-SNR movements.

**Figure 6:**
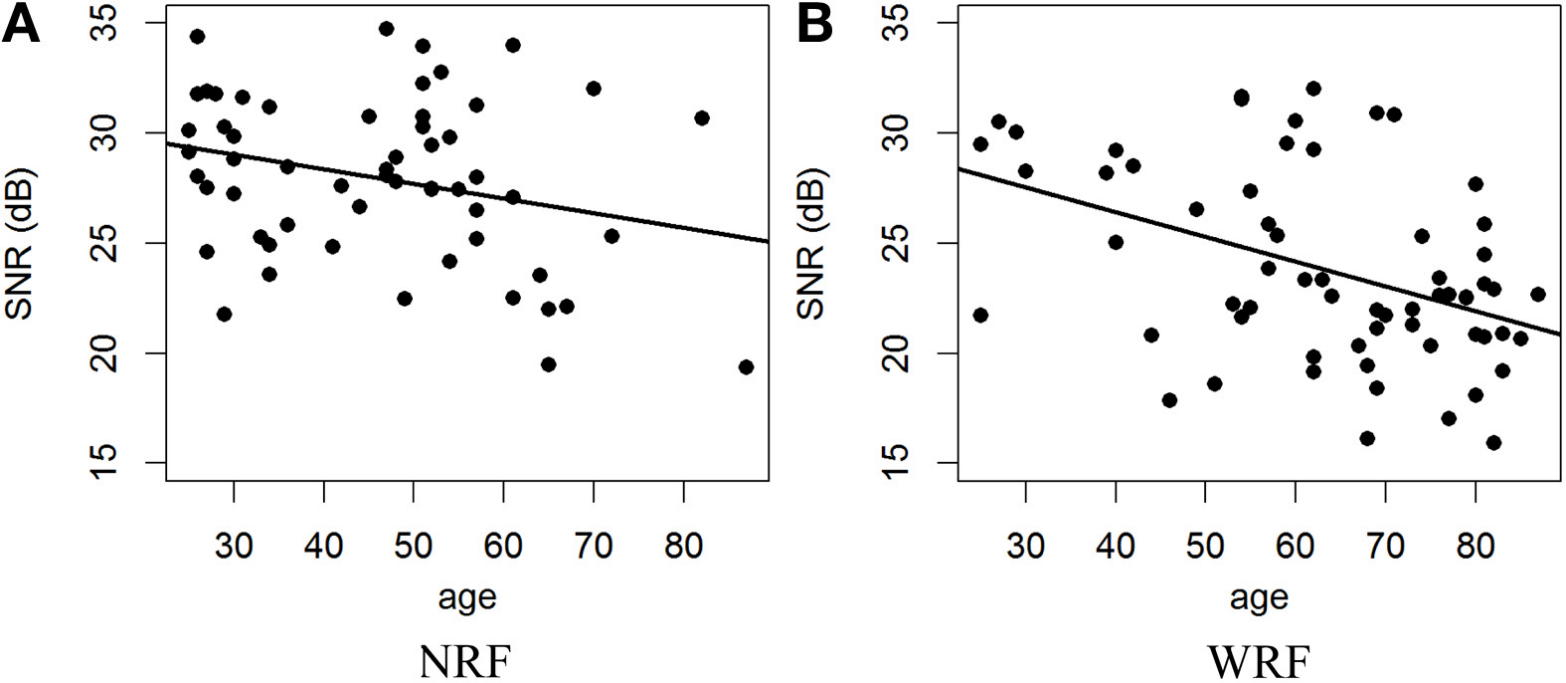
**Scatter plot linking the average SNR to the subjects' age.** The lines show the best linear models associated with these data. The plot **(A)** is for the population with No Risk Factor (NRF) and the plot **(B)** for the population With Risk Factor (WRF).

The relationship between age and the delta-lognormal movement can also be appreciated in Figure 6, which shows a scatter plot of the robust average SNR as function of the subjects' age. To compute this average, SNR from the three reaction time tests was pooled since there were no statistically significant differences between the SNR obtained in these tests (*p* > 0.05). The Minimum Volume Ellipsoid algorithm (Rousseeuw and Leroy, 1987) was used for robust computation.

Using a linear model to regress the SNR toward the age, a significant effect of the age factor is obtained [NRF: *F*_(1, 55)_ = 4.488, *p* = 0.039; WRF: *F*_(1, 61)_ = 14.30, *p* = 0.00038], with a large variability causing a low coefficient of determination (*R*^2^_NFR_ = 0.075, *R*^2^_WFR_ = 0.19). Overall, the effect of age on the deterioration of the motor control causing a decrease of the SNR is supported by these data and the hypothesis of a migration away from a lognormal behavior with the aging process is clearly corroborated.

#### Triangular drawings

A regression analysis similar to the one performed in section Reaching Movements was performed for the data from triangular drawings, with the difference that beside age, the impact of the drawing orientation and triangle size are also modeled^4^. Using α = 0.05 as threshold for statistical significance, only age (*p*_NFR_ = 0.03, *p*_WFR_ = 0.0001) and triangle size (*p*_NFR_ = 0.03, *p*_WFR_ = 0.003 for the difference between large and medium triangles; *p*_NFR_ = 6e−15, *p*_WFR_ < 2−16 for the difference between large and small triangles) were found significant for the SNR, with higher SNR in older subjects and for smaller triangular movements.

For nbLog and SNR/nbLog, only age (*p*_NFR_ = 1e−6, *p*_WFR_ = 8e−11 for nbLog; *p*_NFR_ = 0.0002, *p*_WFR_ = 2e−11 for SNR/nbLog) is significant in both samples. For the WRF sample, the size difference of drawings also has a significant impact when comparing small triangles with large ones for SNR/nbLog (*p*_WFR_ = 0.02).

A second analysis was performed on averaged values of response variables aggregated per subject, regardless of movement properties (i.e., triangle size and orientation). Estimated effects and *p*-values are reported in Table 4. *P*-values are of course less significant for this sigma-lognormal analysis than for the previous regressions on the delta-lognormal data because the sample size is smaller. However, the estimated statistical significances for demographic factors are more reliable because there is no repeated measurement in this analysis.

**Table 4:** **Modeling coefficients and associated p-values computed for the demographic factors**.

**Response**	**NRF**		**WRF**	
**variable**				
	**Intercept (*p*-value)**	**Age (*p*-value)**	**Intercept (*p*-value)**	**Age (*p*-value)**
SNR	**21.28 (5.64e-42)**	0.01516 (0.18)	**21.82 (2.49e-46)**	0.01095 (0.18)
nbLog	**5.889 (3.61e-10)**	**0.03733 (0.037)**	**6.510 (8.41e-6)**	**0.06002 (0.0048)**
SNR/nbLog	**3.499 (2.03e-18)**	−0.009885 (0.079)	**3.196 (1.27e-17)**	**−0.01349 (0.0016)**

Bold is used to indicate statistical significance (p-value <0.05).

As shown in this table, the SNR is not a discriminative factor here. All the subjects were able to carry out the expected task and the ScriptStudio software was able to reconstruct all the trajectories with very high SNR. It is the nbLog, and albeit to a lesser extent SNR/nbLog, that are more relevant. In other words, although the subjects were successful in this specific experiment, this success was obtained at the expense of using more lognormal components to execute their movements.

## Discussion

### Learning

The lognormal performance criteria allowed us to observe a tendency in the young writers to move toward a lognormal behavior as characterized by the three criteria used in this study. In fact, although the participants were early in their learning of the writing process, each of the three criteria (SNR, nbLog, and SNR/nbLog) allow us to observe that the more children advance in their learning, the more their movements tend to have the lognormal scriptural characteristics of better mastered graphomotricity. This phenomenon is more evident with the drawing of oblique traces than for the bridges, which are a more complex shape. From a global and operational perspective, such results can be interesting when considering the definition of rapid tests for the evaluation of young children's motor control abilities in school.

### Aging

The hypothesis that age results in a divergence from lognormality is supported by our study of fast reaching and triangular movements. For the delta-lognormal reconstruction of rapid single strokes, the SNR decrease with age as a result of motor control degradation is generally supported. For the sigma-lognormal reconstruction of the triangles, taking into account the number of lognormal components used to model the movement makes apparent the divergence effect. In other words, the number of lognormal components used in sigma-lognormal modeling is robustly linked to the age factor in this specific experiment. This might suggest that the deterioration of motor control with aging is associated with the development of compensatory strategies such as emitting more motor commands to generate an adequate movement for a given task. One must also take into account that a small part of this effect might be associated with the fact that the Robust *X*_0_ extractor used for obtaining the sigma-lognormal parameters tries to increase the SNR up to 25 dB by adding lognormal components as long as they help to increase the SNR. However, this latter overestimation effect seems to be of mild important since the SNR/nbLog criterion confirms the aging tendency in one of our groups (i.e., WRF).

We also note that, in most cases, the same general trends have been observed in the two samples studied, which gives us high confidence that the relationships reported as significant on both population are not occurring by chance. Put together, the results of section Reaching Movements and Triangular Drawings indicate that lognormality (i.e., high SNR for lognormal modeling) and command efficiency (i.e., small number of lognormal components) are high for young adults and decrease with aging.

### Conclusion

In this paper, we have investigated the concept of the ideal lognormal handwriter, as seen through the paradigm of the Kinematic Theory of rapid human movements. Starting from the fact that this theory predicts a convergence toward a lognormal impulse response for neuromuscular systems that are made up of well-synchronized subsystems, we have extended this interpretation to present the capacity to reconstruct the velocity profile of a movement with lognormal strokes as an indicator of the fine motor control capacity of the person who produced that movement. We first made a brief survey of the Kinematic Theory to clearly define the concept of lognormality and then we reported on some studies demonstrating that lognormality is indeed exploited by mature subjects to succeed in some required tasks. With this ideal descriptor in mind, we then investigated a corollary of this definition: the migration toward lognormality as young children grow up and the deviation from lognormality with aging.

For the first case, we have studied the handwriting of young children. We have shown that three indicators—SNR, nbLog, and SNR/nbLog as extracted from the sigma-lognormal model—can effectively point out the converging behavior toward lognormality of young writers producing simple movements. Indeed, the quality of the reconstruction increases with age and school-based learning of handwriting for oblique traces. For the oblique traces, the older the writer, the better is the control of the movements. In other words, the nbLog is smaller and the SNR/nbLog is higher. To more accurately characterize the PS group in this hierarchy, we will have to increase our dataset and eventually explore other indicators to be combined with those that are already at our disposal.

For the second case, we studied the handwriting of two populations of adults (with and without brain stroke risk factors), each participant being required to produce rapid straight strokes in reaction to a given stimulus and triangular movements. For rapid handwriting strokes, we have shown that the move away from lognormality with age was clearly observed as a decrease of the SNR with age. For triangular movements, the trajectories could be reconstructed with good SNR and the nbLog necessary for reconstruction was clearly associated with the effect of aging, with older participants needing more motor commands to perform the same type of movement.

Apart from using the concept of lognormality to characterize the level of learning in the first years of kindergarten and the effect of aging on human motor control, the same approach can be used to study departure from the ideal lognormal behavior when health problems affect handwriting production. For instance, a complete analysis of the whole population used in this experiment (O'Reilly and Plamondon, 2011, 2012a,b,c; O'Reilly, 2012; Plamondon et al., 2014b) clearly shows that there is a relationship between the presence of brain stroke risk factors and the characteristics of human movements as analyzed with the Kinematic Theory. Although a large part of this may be attributed to the effect of age and gender, there is convincing evidence that these two factors do not account for it all. Furthermore, in a recent study (Van Gemmert et al., 2013), it is observed that the nbLog variable was significantly larger for individuals with Parkinson's disease than for an age-matched control group. A similar analysis on Alzheimer's disease has just started (Impedovo et al., 2013). In this perspective, we can also assume that the same methodology could be used for monitoring the rehabilitation process after some injuries (Rohrer et al., 2002; O'Reilly and Plamondon, 2009b).

### Conflict of interest statement

The authors declare that the research was conducted in the absence of any commercial or financial relationships that could be construed as a potential conflict of interest.

## Footnotes

^1^An exhaustive database of papers in these fields is available at http://www.graphonomics.org/, where the proceedings of all the biennal conferences of the International Graphonomics Society over the last 30 years are deposited.

^2^Although many of these studies were using large strokes and trajectories for practical experimental purposes and to facilitate parameter extraction, the theory has been used for the analysis and the generation of movements of any size, from a few millimeters to decimeter letters, words, or signatures. Since the size of a trajectory is controlled by the parameter D, which determines the stroke amplitude, it has no effect on the lognormal convergence. It must also be noted that the theory is not limited to the control of finger movements. It has been used to analyze wrist, arm and head movements and eye saccades (Plamondon, 1995a) as well as 3D full upper limb planar trajectories (Leduc and Plamondon, 2001).

^3^This threshold is less restrictive than the 25 dB threshold used in the present study because modeling bidirectional primitives with single delta-lognormals generally results in lower SNR.

^4^Using the equation notation used in the S statistical language, the regression performed follows the formula SNR ~ age + size*direction.
